# Draft Genome Sequence and Secondary Metabolite Biosynthetic Potential of the Lysobacter niastensis Type Strain DSM 18481

**DOI:** 10.1128/MRA.01296-20

**Published:** 2021-01-07

**Authors:** Paolo Turrini, Irene Artuso, Marco Tescari, Gabriele Andrea Lugli, Emanuela Frangipani, Marco Ventura, Paolo Visca

**Affiliations:** a Department of Science, Roma Tre University, Rome, Italy; b Laboratory of Probiogenomics, Department of Chemistry, Life Sciences, and Environmental Sustainability, University of Parma, Parma, Italy; c Department of Biomolecular Sciences, University of Urbino Carlo Bo, Urbino, Italy; University of Southern California

## Abstract

Lysobacter niastensis belongs to a group of bacterial predators that produce a number of bioactive small molecules endowed with lytic properties toward other microorganisms. Here, we report the draft genome sequence of the type strain DSM 18481 and the identification of gene clusters implicated in the biosynthesis of secondary metabolites.

## ANNOUNCEMENT

Lysobacter niastensis is an aerobic, rod-shaped, gliding gammaproteobacterium belonging to the *Lysobacteraceae* family (1, 2). The type strain DSM 18481 of *L. niastensis* was isolated from greenhouse soil in the Republic of Korea (2). *Lysobacter* species are bacterial predators endowed with the ability to produce lytic enzymes and peptides capable of causing the death of prokaryotic and eukaryotic microorganisms (3). Despite the limited genetic information available on the genus *Lysobacter*, some strains are emerging sources for novel antibiotics and are amenable for biosynthetic engineering (4, 5). Here, the genome of *L. niastensis* DSM 18481^T^ was sequenced and analyzed for the presence of biosynthetic gene clusters (BGCs) encoding secondary metabolites.

*L. niastensis* DSM 18481^T^ was obtained from the DSMZ and aerobically grown at 28°C in Reasoner’s 2A (R2A) medium. DNA extraction was performed using a QIAamp DNA minikit (Qiagen). A genomic library of *L. niastensis* was obtained with the TruSeq DNA PCR-free sample preparation kit (Illumina, Inc., San Diego, CA, USA). Genome sequencing was performed with a NextSeq 500 sequencing system, according to the supplier’s protocol (Illumina, UK), and library samples were loaded into a midoutput kit v2.5 (300 cycles) (Illumina, UK), producing 1,670,224 paired-end reads. The raw sequence reads were filtered and trimmed using the command-line fastq-mcf software (https://expressionanalysis.github.io/ea-utils/). Fastq files of Illumina paired-end reads (150 bp) were used as input in the MEGAnnotator pipeline for microbial genome assembly and annotation (6). This pipeline employed the program SPAdes v3.14.0 for *de novo* assembly of the genome sequence with the option “--careful” and a list of k-mer sizes of 21, 33, 55, 77, 99, and 127 (7). The genome quality was evaluated with the program CheckM (8), estimating a genome completeness of 99.89% and 0.86% contamination. The contigs were then submitted to the National Center for Biotechnology Information (NCBI) for the prediction of protein-encoding open reading frames (ORFs) and tRNA and rRNA genes using the NCBI Prokaryotic Genome Annotation Pipeline (PGAP) (9). All tools were run with default parameters unless otherwise specified.

The draft genome sequence of *L. niastensis* is 4,034,846 bp long. It was assembled into 15 contigs with an *N*_50_ value of 390,805 bp, an average coverage of 117×, and a mean GC content of 66.88%. Genome annotation identified 3,723 ORFs, 49 tRNA genes, and 3 rRNA genes.

The presence of six BGCs encoding putative secondary metabolites was predicted using the program antiSMASH v5.1.2 (10) (Table 1). Two BGCs were involved in the biosynthesis of putative fatty acids (eicosapentaenoic acid and an arylpolyene), two encoded putative bacteriocins, one was predicted as a hybrid system composed of a nonribosomal peptide synthetase (NRPS) and a type I polyketide synthase (T1PKS), and one was predicted as an NRPS-like cluster. Interestingly, 4 out of 6 BGCs showed no significant similarity with BGCs involved in the synthesis of known compounds, suggesting that their products represent novel secondary metabolites which deserve more in-depth chemical and biosynthetic characterization.

**TABLE 1:** antiSMASH predicted BGCs encoding secondary metabolites in the *Lysobacter niastensis* DSM 18481^T^ draft genome sequence

Contig no.	Nucleotide start–stop position (relative to contig sequence)	Type^*a*^	Closest known cluster(s) (% similarity)	MIBiG accession no.^*b*^	Closest homolog of core biosynthetic gene(s)^*c*^	Species of closest homolog	Identity (%)
2	161495–203387	Resorcinol	Eicosapentaenoic acid (10)	BGC0000865	3-oxoacyl-ACP synthase	*Haliea* sp.	63.43
2	438274–481902	Arylpolyene	Arylpolyene, APE Vf (50)	BGC0000837	Beta-ketoacyl-ACP synthase	Lysobacter ruishenii	91.24
					Beta-ketoacyl-ACP synthase	*Pseudoxanthomonas* sp. strain PXM04	74.36
5	12052–19947	Bacteriocin	ND^*d*^	ND	DUF692 family protein	Lysobacter panacisoli	76.21
5	63268–117003	NRPS/T1PKS	ND	ND	NRPS	*Lysobacter* sp. strain CW239	66.92
					T1PKS	*Lysobacter* sp. strain CW239	61.61
6	254882–297695	NRPS-like	ND	ND	AMP-binding protein	Vulcaniibacterium gelatinicum	67.04
7	135058–145921	Bacteriocin	ND	ND	DUF692 domain-containing protein	Lysobacter capsici	78.95

a The type was defined by antiSMASH analysis; NRPS, nonribosomal peptide synthetase; T1PKS, type 1 polyketide synthase.

b MIBiG, Minimum Information about a Biosynthetic Gene cluster (11); the MIBiG accession number refers to the closest known cluster.

c The core biosynthetic gene was defined by antiSMASH analysis, and the closed homolog was identified by BLASTp interrogation of the NCBI protein database using *L. niastensis* DSM 18481^T^ core biosynthetic gene(s) as query. ACP, acyl carrier protein.

d ND, not determined.

### Data availability.

This whole-genome shotgun project has been deposited at DDBJ/ENA/GenBank under accession number JADLZT000000000. The version described in this paper is JADLZT000000000.1. The raw sequencing reads are available at the Sequence Read Archive under accession number SRR13014585 and are associated with BioProject accession number PRJNA675736.
